# Associations between Inflammatory Cytokine Gene Polymorphisms and Susceptibilities to Intracranial Aneurysm in Chinese Population

**DOI:** 10.1155/2021/8865601

**Published:** 2021-01-16

**Authors:** Lu Xu, Liming Hu, Chongyu Hu, Junyu Liu, Bingyang Li, Xin Liao, Jilin Zhou, Songlin Liu, Yifeng Li, Dun Yuan, Weixi Jiang, Junxia Yan

**Affiliations:** ^1^Department of Neurosurgery, XiangYa Hospital, Central South University, Changsha, China; ^2^Department of Epidemiology and Health Statistics, XiangYa School of Public Health, Central South University, Changsha, China; ^3^Hunan Provincial Key Laboratory of Clinical Epidemiology, XiangYa School of Public Health, Central South University, Changsha, China; ^4^Department of Neurology, Hunan People's Hospital, Changsha, China; ^5^The People's Hospital of Guangxi Zhuang Autonomous Region, Nanning, China

## Abstract

Intracranial aneurysm (IA) is a complex disease caused by genetic and environmental factors. Evidence indicates that inflammation plays an important role in IA occurrence. We aimed to explore the associations between inflammatory cytokine gene polymorphisms and IA in a Chinese population. This study enrolled 768 participants of Han ethnicity, including 384 patients with IA and 384 healthy individuals. Sixteen single nucleotide polymorphisms (SNPs) of *IL1*, *IL6*, *IL12*, and *TNF-α* genes were genotyped using the Sequenom MassARRAY platform. Univariate and multivariate logistic regression analyses were used to analyze the associations. We found *IL12B* rs3181216 was significantly associated with IA in the recessive and additive models (OR = 0.46, 95% CI = 0.23–0.89, *P* = 0.022; OR = 0.74, 95% CI = 0.56–0.98, *P* = 0.034, respectively). *TNF-α* rs1799964 was associated with IA in dominant and additive models (OR = 0.67, 95% CI = 0.46–0.98, *P* = 0.041; OR = 0.71, 95% CI = 0.51–0.98, *P* = 0.034, respectively). *IL1A* rs17561 was associated with single IA susceptibility (dominant model: OR = 0.52, 95% CI = 0.31–0.85, *P* = 0.040). The *IL12B* rs3181216 polymorphism was associated with single IA susceptibility in the recessive model (OR = 0.41, 95% CI = 0.18–0.93, *P* = 0.033). The *IL12B* rs2195940 polymorphism was associated with multiple IAs susceptibility (dominant model: OR = 0.28, 95% CI = 0.09–0.89, *P* = 0.031; additive model: OR = 0.28, 95% CI = 0.09–0.90, *P* = 0.032). *TNF-α* rs1799964 was associated with multiple IAs susceptibility in the dominant model (OR = 0.54, 95% CI = 0.30–0.97, *P* = 0.040). No associations were found between other polymorphisms and IA susceptibility. Therefore, *IL1A*, *IL12B*, and *TNF-α* gene polymorphisms are associated with IA susceptibility in a Chinese population.

## 1. Introduction

Intracranial aneurysm (IA) is a complex disease that affects human health and is characterized by localized abnormal expansion and enlargement of the intracranial artery wall [1]. The prevalence of IA is 3.2% in the middle-aged population [2]. The rupture of IA is the main cause of subarachnoid hemorrhage (SAH), accounting for about 80% of all nontraumatic SAHs, which has a high mortality and disability rate [3, 4]. To date, the pathogenesis, occurrence, growth, and rupture of IA have not yet been clarified. Despite the advancement of medical technology, the prognosis of ruptured IA remains poor. In addition, IA has a negative impact on the society and its individuals [5]. Therefore, it is necessary to identify the factors associated with the occurrence and rupture of IA and prevent the occurrence and development of IA as early as possible.

The etiology of IA is complex. Studies have shown that sex, age, alcohol abuse, hypertension, and genetic factors are the risk factors for IA [6, 7]. Various studies have indicated that inflammatory factors also play important roles in the development of IA. Studies have found that there is a large amount of inflammatory cell infiltration in IA tissues, and the levels of inflammatory cytokines in the cerebrospinal fluid of SAH are significantly increased, but there is no infiltration of inflammatory cells in the normal arterial wall [8–11]. The mechanism of inflammation involved in IA may be related to the inflammation-induced vascular endothelial damage, the degenerative vascular wall remodeling, and a high blood flow, including increased vascular wall shear stress [12, 13]. Candidate gene association studies revealed that polymorphisms in interleukin 1 (*IL1*), interleukin 6 (*IL6*), interleukin 12 (*IL12*), and tumor necrosis factor-*α* (*TNF-α*) genes are associated with IA in several populations [14–17]. Therefore, inflammation may play an important role in the formation of IA, and inflammatory cell infiltration could be considered as a sign of pathological changes in IA. Hence, exploring the relationship between inflammatory cytokines and IA from different aspects will further help us to understand their potential roles in IA.

Although many studies have been conducted on IA-related risk factors, the association between inflammatory cytokine gene polymorphisms and IA remains unclear due to a small sample size and insufficient statistical efficiency [18–20]. In addition, the existence of population heterogeneity may have affected the previous findings. Therefore, in this study, we aimed to explore the associations between inflammatory cytokine gene (*IL1*, *IL6*, *IL12*, and *TNF-α*) polymorphisms and IA susceptibility in a Chinese population.

## 2. Materials and Methods

### 2.1. Study Population

A cohort of IA cases, since January 2016, was recruited from the Department of Neurosurgery and Neurology of Xiangya Hospital and Hunan Provincial People's Hospital. IA was diagnosed using magnetic resonance imaging (MRI), computed tomography angiography (CTA), or digital subtraction angiography (DSA). In parallel, subjects from the same district community health service center who participated in a routine annual health checkup were selected as the control subjects. The subjects included in the control group were those with no history or family history of IA or SAH or other related vascular diseases. All included participants were of Han ethnicity. Demographic and lifestyle information and disease history were collected through a questionnaire survey. Peripheral venous blood was collected from all subjects for reserve. The study was approved by the Medical Ethics Committee of Central South University (CTXY-150002-1), and informed consent was obtained from the study subjects.

### 2.2. Single Nucleotide Polymorphism Selection and Genotyping

Based on the single nucleotide polymorphisms (SNPs) associated with IA found in previous studies and the tagSNPs screened and determined by Genome Variation Server 150 (http://gvs.gs.washington.edu/GVS150/index.jsp), 16 SNPs in *IL1*, *IL6*, *IL12*, and *TNF-α* genes were selected. Genomic DNA extraction from peripheral blood was carried out using the TIANamp Blood Genomic DNA Extraction kit (TIANGEN Biotech Co., Ltd., Beijing, China) according to the manufacturer's instructions. DNA samples were frozen at -80°C until further genotyping.

The SNPs were genotyped using the SEQUENOM MassARRAY platform (Agena Bioscience Inc., San Diego, CA, USA). Primers were designed based on the SNP locus using the Assay Design 3.1 software by Sequenom (Table S1). The PCR reaction was performed as described previously [21]. In brief, the PCR reaction mixture comprised 1 *μ*L template DNA (20–50 ng) and 4 *μ*L PCR Mastermix, which included 1.8 *μ*L dddH_2_O, 0.5 *μ*L 10x PCR Buffer, 0.4 *μ*L MgCl_2_ (25 mM), 0.1 *μ*L dNTP (25 mM), 0.2 *μ*L Hotstar Taq (5 U/*μ*L), and 1 *μ*L PCR Primer mix. The PCR amplification was set up as follows: initial denaturing at 95°C for 2 min, followed by 45 cycles of amplification including denaturing at 95°C for 30 s, annealing at 56°C for 30 s, extension at 72°C for 60 s, and hold at 25°C. After processing the PCR products with the Shrimp alkaline phosphatase, single base extension, and resin desalination reactions, the SNP genotype and allele were identified by matrix-assisted laser desorption/ionization-time of flight (MALDI-TOF) mass spectrometry. The MassArray TYPER 4.0 software was used to detect mass spectrometry peaks, and the genotypes of the target locus in each sample were interpreted according to the mass spectrometry peak patterns.

### 2.3. Statistical Analysis

Statistical analyses were conducted using the SPSS 21.0 software (SPSS Inc., Chicago, IL, USA). Data distribution was evaluated for normality using the Kolmogorov–Smirnov test. Continuous variables were presented as mean ± standard deviation (SD) or median with interquartile range (IQR) based on data distribution. For normally distributed data, Student's *t*-test was used to compare two groups. For nonnormally distributed data, Mann–Whitney *U* test was applied. Categorical variables were expressed as proportions, and the comparison was carried out using *χ*^2^ test or Fisher's exact test. Hardy-Weinberg equilibrium (HWE) of the control groups was tested using *χ*^2^ test to evaluate whether the allele frequency distribution deviated from the HWE. Haploview v.4.2 (https://www.broadinstitute.org/haploview/haploview) was used to estimate pair-wise linkage disequilibrium (LD). Pair-wise LD parameters *r*^2^ and *D*′ were used as criteria for judging LD. If *r*^2^ ≥80%, SNPs were considered in strong LD. At the same time, if *D*′ = 1, SNPs were considered in complete LD [21]. Univariate and multivariate logistic regression analyses were used to detect the association between SNPs and IA, and the odds ratios (ORs) and 95% confidence intervals (CIs) in different models were calculated. *P* < 0.05 was considered statistically significant.

## 3. Results

### 3.1. Characteristics of Study Participants

A total of 384 IA patients and 384 controls were included in this study. There were 284 patients with single IA and 136 patients with multiple IAs. The mean age of the controls was higher than that of the patients. The proportion of hyperlipidemia was higher in the case group, while the proportion of smoking, alcohol consumption, and diabetes was higher in the control group. The aneurysms were primarily located in the internal carotid artery, followed by the middle cerebral artery, and other locations. The detailed demographic and clinical characteristics of the study participants are described in Table 1.

### 3.2. Hardy-Weinberg Equilibrium and Linkage Disequilibrium Analysis

**Figure 1:**
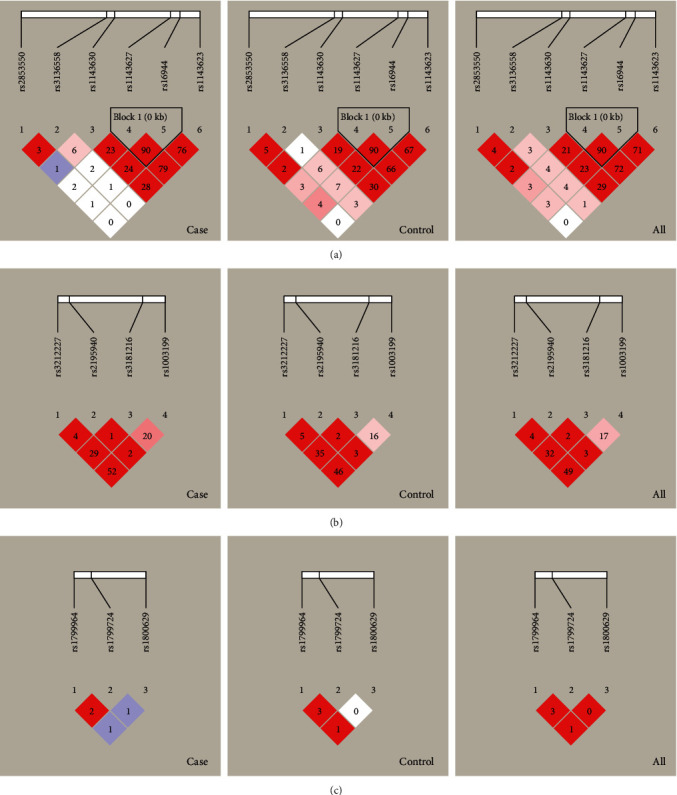
Linkage disequilibriums of *IL1B* (a), *IL12B* (b), and *TNF-α* (c) in case, control, and total groups.

All SNPs had a 100% genotyping call rate, and the risk allele frequencies were more than 4% in the participants. The genotype distributions of all SNPs were in HWE in the normal control group (*P* > 0.05) (Table 2). LD analysis showed that the SNPs of *IL1B* (rs1143627 and rs16944) were in strong LD (*r*^2^ > 0.8) (Figure 1). We chose to focus on the SNP rs1143627.

### 3.3. Associations between SNPs of Inflammatory Cytokine Genes and Total IA

The genotype distribution of the SNPs is shown in Table 2. *IL1A* rs17561 and *IL1B* rs2853550 polymorphisms were significantly associated with IA in univariate logistic regression analysis (Table S2). However, the associations between *IL1A* and *IL1B* polymorphisms and IA were not significant after adjusting for age, smoking, drinking, diabetes, and hyperlipidemia status (Table 2). In multivariate logistic regression analysis, a significant association was found between rs3181216 in *IL12B* and IA (recessive model: OR = 0.46, 95% CI = 0.23-0.89, *P* = 0.022; additive model: OR = 0.74, 95% CI = 0.56-0.98, *P* = 0.034). *TNF-α* rs1799964 was associated with IA susceptibility (dominant model: OR = 0.67, 95% CI = 0.46-0.98, *P* = 0.041; additive model: OR = 0.71, 95% CI = 0.51-0.98, *P* = 0.034) (Table 2). No association was found between *IL1B* rs1143627, rs16944, rs1143623, rs1143630, rs3136558; *IL6* rs1800795, rs1800796; *IL12B* rs3212227, rs1003199, rs2195940; and *TNF-α* rs1800629 rs1799724 polymorphisms and IA susceptibility in any genetic model in either univariate or multivariate logistic regression analysis.

### 3.4. Associations between SNPs of Inflammatory Cytokine Genes and Single IA

The genotype distribution of SNPs for single IA is shown in Table 3. *IL1A* rs17561 and *IL1B* rs2853550 polymorphisms were significantly associated with single IA in univariate logistic regression analysis (Table S3). However, the associations between *IL1B* polymorphisms and single IA were not significant after adjusting for age, smoking, drinking, diabetes, and hyperlipidemia status. In multivariate logistic regression analysis, *IL1A* rs17561 was associated with the formation of single IA (dominant model: OR = 0.52, 95% CI = 0.31-0.85, *P* = 0.040). The *IL12B* rs3181216 polymorphism was associated with single IA in the recessive model (OR = 0.41, 95% CI = 0.18-0.93, *P* = 0.033) (Table 3). No association was found between other polymorphisms and single IA.

### 3.5. Associations between SNPs of Inflammatory Cytokine Genes and Multiple IAs

The genotype distribution of SNPs for multiple IAs is shown in Table 4. *IL12B* rs2195940 polymorphism was associated with multiple IAs (dominant model: OR = 0.28, 95% CI = 0.09-0.89, *P* = 0.031; additive model: OR = 0.28, 95% CI = 0.09-0.90, *P* = 0.032) in multivariate logistic regression analysis. *TNF-α* rs1799964 was associated with multiple IAs in both univariate (Table S4) and multivariate logistic regression analysis (dominant model: OR = 0.54, 95% CI = 0.30-0.97, *P* = 0.040) (Table 4). No association was found between other polymorphisms and multiple IAs.

## 4. Discussion

In this study, we investigated the associations between SNPs of inflammatory cytokine genes and IA risk in a Chinese population. The results showed that *IL12B* rs3181216 and *TNF-α* rs1799964 were associated with total IA. *IL1A* rs17561 and *IL12B* rs3181216 polymorphisms were associated with single IA, while *IL12B* rs2195940 and *TNF-α* rs1799964 polymorphisms were associated with multiple IAs.

*IL1* is a cytokine mainly produced by activated macrophages, which can stimulate the proliferation and differentiation of cells involved in immune response and improve their function. The *IL1* family consists of three homologous proteins: IL1A, IL1B, and IL1 receptor antagonist (IL1RA), which are encoded by the three genes *IL1A*, *IL1B*, and *IL1RA*, respectively, located on the chromosome region 2q12-q21 [22]. *IL1* often has abnormal expression in the pathological process of infection, injury, and inflammation, which can also affect the occurrence, development, and prognosis of multiple sclerosis, Parkinson's disease, epilepsy, Alzheimer's disease, tumor, and immune diseases [23]. Slowik et al. found that *IL1B* gene polymorphism can increase the risk of aneurysmal SAH in Poland [14]. However, no association was identified between rs16944 and rs1143627 of *IL1B* and IA in Italy and Poland, respectively [24, 25]. In this study, no associations were observed between *IL1B* gene polymorphisms (rs1143627, rs16944, 1143623, rs1143630, rs2853550, and rs3136558) and IA in multivariate logistic regression analysis after adjusting for confounding factors. However, we found that *IL1A* rs17561 was associated with single IA. The difference in results may be due to population heterogeneity. In addition, inconsistent conclusions were drawn in univariate and multivariate logistic regression analyses in this study, and therefore, more research is needed to further discuss the associations between *IL1* gene polymorphisms and IA. A study has shown that neutrophil extracellular traps induced by *IL1B* play a major role in the formation of abdominal aortic aneurysms [26]. Although there are many similarities between IA and abdominal aortic aneurysm, and they share common risk factors, such as smoking and hypertension [7, 27], the associations between *IL1* gene polymorphisms and IA susceptibility still lack consistent conclusions. The role of *IL1* in IA risk is still unclear. Therefore, the association between *IL1* gene polymorphisms and IA and its mechanism needs to be studied in the future.

*IL12*, a cytokine with a wide range of biological activities, is mainly produced by activated inflammatory cells [28]. The active *IL12* cytokine is a 70-kDa heterodimer composed of two chains of p35 (*IL12A*) and p40 (*IL12B*) [16, 29]. *IL12B* is located on chromosome 5q33.3. A study found that the *IL12B* rs3212227 polymorphism is associated with IA susceptibility in the Chinese population [16], but the association was not found in the Indian population [25]. In this study, we did not find an association between rs3212227 and IA, which is consistent with previous meta-analysis results [25]. No association was found between *IL12B* rs1003199 and IA risk. However, we found that *IL12B* rs3181216 was associated with single IA, and *IL12B* rs2195940 was associated with multiple IAs, indicating that these SNPs may be new potential risk loci for IA in the Chinese population. Studies have indicated that *IL12B* is associated with many immune-mediated inflammatory diseases [30, 31], and inflammation plays an important role in the formation of IA [32]. Based on these results, it can be considered that *IL12B* gene polymorphism may be associated with IA, but the specific loci and mechanism still need further study.

*TNF-α* is the primary proinflammatory cytokine that triggers inflammation and immune response [33]. The human *TNF-α* gene is located on the short arm of chromosome 6 (6p21.32) [34]. A study found that *TNF-α* rs1799964 is associated with an increased risk of IA in the Chinese population [35]. Consistently, we identified that the rs1799964 polymorphism was significantly associated with susceptibility to multiple IAs. The increase in *TNF-α* expression coincides with the growth and rupture of IA, and the inhibition of *TNF-α* can reduce the occurrence and rupture of IA [36], confirming the association between *TNF-α* and IA. In accordance with the results of a previous study [35], we did not find any association between rs1799724 of *TNF-α* and IA. In addition, we did not find an association between rs1800629 and IA in this study, which is contrary to a study conducted in Brazil [17]. This may be due to the different genetic susceptibilities of different populations. It has been pointed out that the involvement of *TNF-α* in IA may be related to its promotion of inflammatory response and subsequent weakening of the blood vessel wall caused by apoptosis of blood vessels and immune cells [37]. However, the mechanism of *TNF-α* and IA is not fully understood, and future studies are needed.

*IL6* is a pleiotropic inflammatory cytokine with proinflammatory effects that can stimulate the growth of mature B cells and promote the synthesis of C-reactive protein in the process of tissue injury [38]. The human *IL6* gene is located on chromosome 7 and encodes a product of 212 amino acids [39]. Studies have indicated that *IL6* gene promoter polymorphisms are associated with IA in Chinese and British populations [19, 20, 40, 41]. In contrast, we did not find any associations between *IL6* gene polymorphisms (rs1800795 and rs1800796) and IA in this study, which is consistent with the results of Bayri and Pera et al. [18, 42]. The inconsistent results may be because of genetic heterogeneity and different environmental factors in different populations. Our results were different from previous studies in the Chinese population [19, 20, 40]; some of the differences may be explained by the fact that the age of the study subjects in our study was higher than that in previous studies conducted in China, and more research is needed to discuss the associations.

Some limitations of the current study should be noted. First, the controls included in this study were individuals recruited from the community who had no history of IA or other cerebrovascular diseases and family history, but without confirmation of IA disease absence by clinical imaging; in terms of age, the control group was older than the case group, which may have some impact on the results. However, considering that the controls were not diagnosed with IAs or other cerebrovascular diseases during the investigation period, they should also have been healthy 10 years ago; therefore, we believe that the results of this study were reliable. Second, significant differences in the proportions of smoking, drinking, diabetes, and hyperlipemia between patients and controls may also affect the outcomes, and thus, the conclusions. However, we used multivariate logistic regression analysis to analyze these factors, and hence, we believe that our results have a certain degree of credibility. Third, we did not study the function of IA-related loci, and the mechanism of action of inflammatory cytokine gene polymorphisms in the occurrence of IA remains unclear. Fourth, the cases in our study have limited sources, and the association between inflammatory cytokine gene polymorphisms and IA requires further study.

## 5. Conclusions

Our results indicated that *IL1A* rs17561 and *IL12B* rs3181216 polymorphisms were associated with single IA and *IL12B* rs2195940 and *TNF-α* rs1799964 polymorphisms were associated with multiple IAs in a Chinese population. These findings can help us better understand the genetic risk factors of IA. Further studies are needed to confirm the associations and pathological mechanisms of IA.

## Figures and Tables

**Table 1:** Characteristics of the study participants.

Variables	IA group			
	Total (*n* = 384)	Single IA (*n* = 248)	Multiple IAs (*n* = 136)	Control group (*n* = 384)
Mean age, year (SD)	57.1 ± 10.6^∗^	57.3 ± 10.4^∗^	56.6 ± 10.9^∗^	66.5 ± 2.1
Female, *n* (%)	267 (69.5)	166 (66.9)	101 (74.3)	267 (69.5)
Smoking, *n* (%)	59 (15.4)^∗^	42 (16.9)^∗^	17 (12.5)^∗^	111 (28.9)
Drinking, *n* (%)	36 (9.4)^∗^	24 (9.7)^∗^	12 (8.8)^∗^	77 (20.1)
Hypertension, *n* (%)	208 (54.2)	127 (51.2)	81 (59.6)	200 (52.1)
Diabetes, *n* (%)	24 (6.3)^∗^	15 (6.0)^∗^	9 (6.6)^∗^	65 (16.9)
Hyperlipemia, *n* (%)	22 (5.7)^∗^	13 (5.2)^∗^	9 (6.6)^∗^	8 (2.1)
Site of intracranial aneurysm, *n* (%)				
Internal carotid artery	287 (48.7)	113 (45.6)	174 (51.0)	—
Middle cerebral artery	114 (19.4)	45 (18.1)	69 (20.2)	—
Anterior communicating artery	61 (10.4)	41 (16.5)	20 (5.9)	—
Anterior cerebral artery	39 (6.6)	15 (6.1)	24 (7.1)	—
Posterior communicating artery	34 (5.8)	18 (7.3)	16 (4.7)	—
Basilar artery	30 (5.1)	14 (5.6)	16 (4.7)	—
Posterior cerebral artery	15 (2.5)	2 (0.8)	13 (3.8)	—
Other	9 (1.5)	—	9 (2.6)	—

^∗^Significant in comparison with control (*P* < 0.05); SD: standard deviation.

**Table 2:** Multivariate logistic regression analysis of associations between inflammatory cytokine gene polymorphisms and risk of IA in Chinese population.

Gene	SNPs	Genotype		Dominant model		Recessive model		Additive model		*P*_HWE_^†^
		Case (*n*)	Control (*n*)	OR (95% CI)	*P*	OR (95% CI)	*P*	OR (95% CI)	*P*	
*IL-1A*	rs17561C>A	341/41/2	318/65/1	0.68 (0.41-1.12)	0.132	1.54 (0.10-23.79)	0.757	0.71 (0.44-1.15)	0.163	0.218
*IL-1B*	rs1143627G>A	77/199/108	93/185/106	1.28 (0.83-1.96)	0.267	1.09 (0.73-1.61)	0.677	1.13 (0.88-1.45)	0.359	0.489
	rs16944A>G	71/196/117	88/187/109	1.33 (0.86-2.08)	0.204	1.19 (0.81-1.74)	0.385	1.18 (0.92-1.52)	0.195	0.651
	rs1143623C>G	134/185/65	144/179/61	1.00 (0.69-1.44)	0.986	0.93 (0.57-1.52)	0.773	0.98 (0.76-1.26)	0.870	0.666
	rs1143630T>G	12/108/264	13/102/269	1.37 (0.51-3.68)	0.538	1.09 (0.74-1.61)	0.657	1.10 (0.79-1.53)	0.559	0.391
	rs2853550A>G	0/58/326	5/75/304	—	—	1.23 (0.79-1.94)	0.363	1.31 (0.85-2.01)	0.220	0.878
	rs3136558A>G	160/176/48	171/172/41	0.91 (0.63-1.30)	0.587	0.84 (0.48-1.49)	0.559	0.91 (0.70-1.19)	0.493	0.817
*IL-6*	rs1800795C>G	0/0/384	0/0/384	—	—	—	—	—	—	—
	rs1800796G>C	17/137/230	18/141/225	1.27 (0.55-2.97)	0.579	0.98 (0.69-1.41)	0.918	1.02 (0.75-1.38)	0.909	0.491
*IL-12B*	rs3181216A>T	207/152/25	191/155/38	0.77 (0.54-1.10)	0.147	0.46 (0.23-0.89)	0.022	0.74 (0.56-0.98)	0.034	0.429
	rs3212227T>G	105/191/88	107/195/82	1.00 (0.67-1.48)	0.994	1.13 (0.74-1.72)	0.570	1.04 (0.81-1.34)	0.741	0.696
	rs1003199C>T	131/208/45	148/190/46	1.24 (0.86-1.80)	0.253	1.02 (0.59-1.78)	0.932	1.13 (0.86-1.49)	0.372	0.205
	rs2195940C>T	354/28/2	341/41/2	0.55 (0.30-1.04)	0.065	0.76 (0.06-9.50)	0.828	0.59 (0.33-1.06)	0.079	0.528
*TNF-α*	rs1800629G>A	337/46/1	342/41/1	1.14 (0.67-1.96)	0.632	1.48 (0.04-54.63)	0.831	1.14 (0.68-1.93)	0.620	0.844
	rs1799724C>T	293/86/5	298/77/9	1.02 (0.67-1.54)	0.944	0.60 (0.16-2.23)	0.444	0.97 (0.67-1.40)	0.868	0.141
	rs1799964T>C	272/102/10	252/112/20	0.67 (0.46-0.98)	0.041	0.58 (0.24-1.41)	0.230	0.71 (0.51-0.98)	0.034	0.111

SNPs: single nucleotide polymorphisms; OR: odds ratio; CI: confidence interval; HWE: Hardy-Weinberg equilibrium. ^∗^Genotype presented as wild type/heterozygous/homozygous, ^†^HWE *P* value for the control group.

**Table 3:** Multivariate logistic regression analysis of associations between inflammatory cytokine gene polymorphisms and risk of single IA in Chinese population.

Gene	SNPs	Genotype^∗^		Dominant model		Recessive model		Additive model		*P*_HWE_^†^
		Case (*n*)	Control (*n*)	OR (95% CI)	*P*	OR (95% CI)	*P*	OR (95% CI)	*P*	
*IL-1A*	rs17561	224/23/1	318/65/1	0.52 (0.28-0.97)	0.040	2.37 (0.15-38.57)	0.543	0.57 (0.32-1.03)	0.064	0.218
*IL-1B*	rs1143627	48/126/74	93/185/106	1.18 (0.72-1.93)	0.523	1.21 (0.77-1.90)	0.400	1.14 (0.86-1.53)	0.362	0.489
	rs16944	44/128/76	88/187/109	1.28 (0.77-2.15)	0.340	1.22 (0.78-1.90)	0.387	1.18 (0.88-1.58)	0.265	0.651
	rs1143623	91/115/42	144/179/61	0.92 (0.60-1.40)	0.683	1.09 (0.62-1.90)	0.770	0.98 (0.73-1.32)	0.895	0.666
	rs1143630	8/68/172	13/102/269	1.47 (0.45-4.75)	0.524	1.01 (0.65-1.57)	0.971	1.05 (0.72-1.53)	0.805	0.391
	rs2853550	0/35/213	5/75/304	—	—	1.56 (0.91-2.69)	0.110	1.61 (0.96-2.71)	0.074	0.878
	rs3136558	99/115/34	171/172/41	1.05 (0.70-1.59)	0.812	0.93 (0.48-1.77)	0.813	1.01 (0.74-1.38)	0.949	0.817
*IL6*	rs1800795	0/0/248	0/0/384	—	—	—	—	—	—	—
	rs1800796	10/84/154	18/141/225	1.37 (0.50-3.76)	0.537	1.07 (0.71-1.62)	0.747	1.09 (0.77-1.55)	0.621	0.491
*IL12B*	rs3181216	136/97/15	191/155/38	0.80 (0.53-1.20)	0.274	0.41 (0.18-0.93)	0.033	0.75 (0.54-1.03)	0.073	0.429
	rs3212227	77/116/55	107/195/82	0.89 (0.57-1.38)	0.593	1.04 (0.64-1.70)	0.862	0.97 (0.73-1.28)	0.811	0.696
	rs1003199	84/130/34	148/190/46	1.11 (0.73-1.71)	0.621	0.88 (0.46-1.69)	0.706	1.03 (0.75-1.41)	0.855	0.205
	rs2195940	226/20/2	341/41/2	0.71 (0.35-1.43)	0.340	1.00 (0.08-12.85)	0.998	0.75 (0.40-1.43)	0.383	0.528
*TNF-α*	rs1800629	218/29/1	342/41/1	1.27 (0.69-2.35)	0.443	2.12 (0.05-85.22)	0.691	1.27 (0.71-2.30)	0.422	0.844
	rs1799724	187/60/1	298/77/9	0.95 (0.59-1.53)	0.824	0.03 (0.00-2.93)	0.135	0.86 (0.55-1.33)	0.488	0.141
	rs1799964	168/75/5	252/112/20	0.70 (0.45-1.09)	0.116	0.38 (0.11-1.26)	0.113	0.70 (0.48-1.02)	0.060	0.111

SNPs: single nucleotide polymorphisms; OR: odds ratio; CI: confidence interval; HWE: Hardy-Weinberg equilibrium. ^∗^Genotype presented as wild type/heterozygous/homozygous. ^†^HWE *P* value for the control group.

**Table 4:** Multivariate logistic regression analysis of associations between inflammatory cytokine gene polymorphisms and risk of multiple IAs in Chinese population.

Gene	SNPs	Genotype^∗^		Dominant model		Recessive model		Additive model		*P*_HWE_^†^
		Case (*n*)	Control (*n*)	OR (95% CI)	*P*	OR (95% CI)	*P*	OR (95% CI)	*P*	
*IL-1A*	rs17561	117/18/1	318/65/1	1.18 (0.59-2.36)	0.635	0.33 (0.00-107.13)	0.710	1.15 (0.59-2.26)	0.681	0.218
*IL-1B*	rs1143627	29/73/34	93/185/106	1.23 (0.64-2.38)	0.534	0.93 (0.52-1.68)	0.806	1.04 (0.71-1.52)	0.835	0.489
	rs16944	27/68/41	88/187/109	1.25 (0.64-2.45)	0.512	1.20 (0.68-2.11)	0.537	1.16 (0.80-1.70)	0.434	0.651
	rs1143623	43/70/23	144/179/61	1.16 (0.67-2.02)	0.594	0.84 (0.39-1.80)	0.654	1.03 (0.70-1.51)	0.887	0.666
	rs1143630	4/40/92	13/102/269	1.21 (0.29-4.99)	0.795	1.24 (0.69-2.22)	0.479	1.19 (0.73-1.95)	0.494	0.391
	rs2853550	0/23/113	5/75/304	—	—	0.75 (0.39-1.42)	0.371	0.84 (0.47-1.52)	0.568	0.878
	rs3136558	61/61/14	171/172/41	0.67 (0.39-1.14)	0.137	0.65 (0.26-1.61)	0.347	0.72 (0.47-1.09)	0.118	0.817
*IL6*	rs1800795	0/0/136	0/0/384	—	—	—	—	—	—	—
	rs1800796	7/53/76	18/141/225	1.10 (0.32-3.73)	0.879	0.88 (0.52-1.49)	0.640	0.93 (0.60-1.44)	0.737	0.491
*IL12B*	rs3181216	71/55/10	191/155/38	0.68 (0.40-1.15)	0.150	0.58 (0.23-1.47)	0.249	0.72 (0.48-1.08)	0.109	0.429
	rs3212227	28/75/33	107/195/82	1.21 (0.66-2.22)	0.532	1.32 (0.72-2.43)	0.369	1.20 (0.82-1.74)	0.352	0.696
	rs1003199	47/78/11	148/190/46	1.35 (0.76-2.38)	0.307	1.10 (0.49-2.48)	0.814	1.20 (0.80-1.81)	0.381	0.205
	rs2195940	128/8/0	341/41/2	0.28 (0.09-0.89)	0.031	—	—	0.28 (0.09-0.90)	0.032	0.528
*TNF-α*	rs1800629	119/17/0	342/41/1	1.05 (0.47-2.36)	0.910	—	—	1.04 (0.47-2.32)	0.923	0.844
	rs1799724	106/26/4	298/77/9	1.16 (0.64-2.09)	0.624	1.79 (0.44-7.26)	0.417	1.19 (0.72-1.96)	0.493	0.141
	rs1799964	104/27/5	252/112/20	0.54 (0.30-0.97)	0.040	0.95 (0.30-2.96)	0.928	0.67 (0.41-1.07)	0.095	0.111

SNPs: single nucleotide polymorphisms; OR: odds ratio; CI: confidence interval; HWE: Hardy-Weinberg equilibrium. ^∗^Genotype presented as wild type/heterozygous/homozygous. ^†^HWE *P* value for the control group.

## Data Availability

The data used to support the findings of this study are included within the article and supplementary information files.

## Supplementary Materials

Supplementary Materials Table S1: PCR primers designed for SNPs. Table S2: univariate logistic regression analysis of associations between inflammatory cytokine gene polymorphisms and risk of IA in Chinese population. Table S3: univariate logistic regression analysis of associations between inflammatory cytokine gene polymorphisms and risk of single IA in Chinese population. Table S4: univariate logistic regression analysis of associations between inflammatory cytokine gene polymorphisms and risk of multiple IAs in Chinese population.
