# Supplementary material for: Associations between Inflammatory Cytokine Gene Polymorphisms and Susceptibilities to Intracranial Aneurysm in Chinese Population
Source: Biomed Res Int. 2021 Jan 16;2021:8865601. doi: 10.1155/2021/8865601 (PMC7826207; doi:10.1155/2021/8865601)
Supplement: Supplementary Materials — Table S1: PCR primers designed for SNPs. Table S2: univariate logistic regression analysis of associations between inflammatory cytokine gene polymorphisms and risk of IA in Chinese population. Table S3: univariate logistic regression analysis of associations between inflammatory cytokine gene polymorphisms and risk of single IA in Chinese population. Table S4: univariate logistic regression analysis of associations between inflammatory cytokine gene polymorphisms and risk of multiple IAs in Chinese population. [file 8865601.f1.zip › Table S1 (2).docx]

| **Table S1.** PCR primers designed for SNPs | | | | |
| --- | --- | --- | --- | --- |
| SNPs | Gene | Primer | | Length (bp) |
| rs17561 | *IL1A* | Forward | 5’-ATACTTTGATTGAGGGCGTCA-3’ | 109 |
|  |  | Reverse | 5’-TCATCAAGCCTAGGTCAGCA-3’ |  |
| rs1143627 | *IL1B* | Forward | 5’-ATCTGCCAGTTTCTCCCTCG-3’ | 124 |
|  |  | Reverse | 5’-ACCAATACTCTTTTCCCCTTTCC-3’ |  |
| rs16944 | *IL1B* | Forward | 5’-GGGTACAATGAAGGGCCAATAG-3’ | 100 |
|  |  | Reverse | 5’-TGCAATTGACAGAGAGCTCC-3’ |  |
| rs1143623 | *IL1B* | Forward | 5’-TGCTTGAATGGGTGAATGGG-3’ | 124 |
|  |  | Reverse | 5’-TCCCTCGTGTCTCAAATACTTG-3’ |  |
| rs1143630 | *IL1B* | Forward | 5’-AGATTATCCCTCTCTGAAGCTCA-3’ | 102 |
|  |  | Reverse | 5’-AGGTGTCAGAAAGCCCACAT-3’ |  |
| rs2853550 | *IL1B* | Forward | 5’-ACTCCCTGCAGTGCTTCAG-3’ | 114 |
|  |  | Reverse | 5’-TATCCTTGGCCACCGAAGAC-3’ |  |
| rs3136558 | *IL1B* | Forward | 5’-CTCAGAGAGGAGGAAAGGGC-3’ | 131 |
|  |  | Reverse | 5’-TGACCTAAATGATTCATCCACCT-3’ |  |
| rs1800795 | *IL6* | Forward | 5’-GCTAGCCTCAATGACGACCT-3’ | 108 |
|  |  | Reverse | 5’-GGTGGGGCTGATTGGAAAC-3’ |  |
| rs1800796 | *IL6* | Forward | 5’-AGTCACACACTCCACCTGG-3’ | 151 |
|  |  | Reverse | 5’-CAAGCCTGGGATTATGAAGAAGG-3’ |  |
| rs3181216 | *IL12B* | Forward | 5’-TAGAAGGATGGGCAGAACTCC-3’ | 142 |
|  |  | Reverse | 5’-TAGTATTACTATTTTGGTTGT-3’ |  |
| rs3212227 | *IL12B* | Forward | 5’-GCAACTTGAGAGCTGGAAAATC-3’ | 162 |
|  |  | Reverse | 5’-ACACAACGGAATAGACCCAAAA-3’ |  |
| rs1003199 | *IL12B* | Forward | 5’-CAAAGGCTTGTTGGGAAAGG-3’ | 169 |
|  |  | Reverse | 5’-ACCTCTGTGCACATTTCCCT-3’ |  |
| rs2195940 | *IL12B* | Forward | 5’-TGTCTTAGGTTCTCTGTGTCTGT-3’ | 135 |
|  |  | Reverse | 5’-ATTGAGTGCTTCCTATGTGCT-3’ |  |
| rs1800629 | *TNF-α* | Forward | 5’-GGCAATAGGTTTTGAGGGGC-3’ | 116 |
|  |  | Reverse | 5’-ACACTCCCCATCCTCCCTG-3’ |  |
| rs1799724 | *TNF-α* | Forward | 5’-CAGCAATGGGTAGGAGAATGTC-3’ | 156 |
|  |  | Reverse | 5’-TGTGGCCATATCTTCTTAAACGT-3’ |  |
| rs1799964 | *TNF-α* | Forward | 5’-CTCAGAGAGCTTCAGGGATATG-3’ | 182 |
|  |  | Reverse | 5’-GGTCTCCTGTAACCCATTCCT-3’ |  |
| SNPs, single nucleotide polymorphisms. | | | | |
