# Supplementary material for: Associations between Inflammatory Cytokine Gene Polymorphisms and Susceptibilities to Intracranial Aneurysm in Chinese Population
Source: Biomed Res Int. 2021 Jan 16;2021:8865601. doi: 10.1155/2021/8865601 (PMC7826207; doi:10.1155/2021/8865601)
Supplement: Supplementary Materials — Table S1: PCR primers designed for SNPs. Table S2: univariate logistic regression analysis of associations between inflammatory cytokine gene polymorphisms and risk of IA in Chinese population. Table S3: univariate logistic regression analysis of associations between inflammatory cytokine gene polymorphisms and risk of single IA in Chinese population. Table S4: univariate logistic regression analysis of associations between inflammatory cytokine gene polymorphisms and risk of multiple IAs in Chinese population. [file 8865601.f1.zip › Table S2 (2).docx]

| **Table S2.** Univariate logistic regression analysis of associations between inflammatory cytokine gene polymorphisms and risk of intracranial aneurysm in Chinese population | | | | | | | | | | |
| --- | --- | --- | --- | --- | --- | --- | --- | --- | --- | --- |
| Gene | SNPs | Genotype* | | Dominant model | | Recessive model | | Additive model | | *P*_HWE_^†^ |
|  |  | Case (n) | Control (n) | OR (95% CI) | *P* | OR (95% CI) | *P* | OR (95% CI) | *P* |  |
| *IL-1A* | rs17561 | 341/41/2 | 318/65/1 | 0.61(0.40-0.92) | 0.018 | 2.01(0.18-22.21) | 0.571 | 0.65(0.43-0.96) | 0.030 | 0.218 |
| *IL-1B* | rs1143627 | 77/199/108 | 93/185/106 | 1.27(0.91-1.79) | 0.165 | 1.03(0.75-1.41) | 0.872 | 1.10(0.90-1.34) | 0.357 | 0.489 |
|  | rs16944 | 71/196/117 | 88/187/109 | 1.31(0.92-1.86) | 0.131 | 1.11(0.81-1.51) | 0.527 | 1.14(0.93-1.40) | 0.200 | 0.651 |
|  | rs1143623 | 134/185/65 | 144/179/61 | 1.12(0.83-1.50) | 0.453 | 1.08(0.74-1.58) | 0.697 | 1.08(0.88-1.32) | 0.469 | 0.666 |
|  | rs1143630 | 12/108/264 | 13/102/269 | 1.09(0.49-2.41) | 0.839 | 0.94(0.69-1.28) | 0.695 | 0.97(0.74-1.26) | 0.788 | 0.391 |
|  | rs2853550 | 0/58/326 | 5/75/304 | 11.15(0.61-202.25) | 0.103 | 1.48(1.02-2.15) | 0.039 | 1.54(1.08-2.20) | 0.017 | 0.878 |
|  | rs3136558 | 160/176/48 | 171/172/41 | 1.12(0.85-1.50) | 0.423 | 1.20(0.77-1.86) | 0.430 | 1.11(0.90-1.37) | 0.332 | 0.817 |
| *IL6* | rs1800795 | 0/0/384 | 0/0/384 | - | - | - | - | - | - | - |
|  | rs1800796 | 17/137/230 | 18/141/225 | 1.06(0.54-2.09) | 0.863 | 1.06(0.79-1.41) | 0.713 | 1.05(0.82-1.34) | 0.710 | 0.491 |
| *IL12B* | rs3181216 | 207/152/25 | 191/155/38 | 0.85(0.64-1.12) | 0.248 | 0.63(0.38-1.07) | 0.090 | 0.83(0.67-1.04) | 0.103 | 0.429 |
|  | rs3212227 | 105/191/88 | 107/195/82 | 1.03(0.75-1.41) | 0.872 | 1.10(0.78-1.54) | 0.602 | 1.04(0.85-1.28) | 0.681 | 0.696 |
|  | rs1003199 | 131/208/45 | 148/190/46 | 1.21(0.90-1.63) | 0.202 | 0.98(0.63-1.51) | 0.911 | 1.10(0.89-1.37) | 0.374 | 0.205 |
|  | rs2195940 | 354/28/2 | 341/41/2 | 0.67(0.41-1.10) | 0.111 | 1.00(0.14-7.14) | 1.000 | 0.71(0.45-1.12) | 0.142 | 0.528 |
| *TNF-α* | rs1800629 | 337/46/1 | 342/41/1 | 1.14(0.73-1.77) | 0.573 | 1.00(0.06-16.05) | 1.000 | 1.13(0.73-1.73) | 0.586 | 0.844 |
|  | rs1799724 | 293/86/5 | 298/77/9 | 1.08(0.77-1.51) | 0.668 | 0.55(0.18-1.66) | 0.287 | 1.01(0.75-1.37) | 0.939 | 0.141 |
|  | rs1799964 | 272/102/10 | 252/112/20 | 0.79(0.58-1.07) | 0.121 | 0.49(0.23-1.05) | 0.068 | 0.78(0.60-1.00) | 0.052 | 0.111 |
| SNPs, single nucleotide polymorphisms; OR, odds ratio; CI, confidence interval; HWE, Hardy-Weinberg equilibrium. | | | | | | | | | | |
| *Genotype presented as wild type/heterozygous/homozygous, † HWE *P* value for the control group. | | | | | | | | | | |
