# Supplementary material for: Associations between Inflammatory Cytokine Gene Polymorphisms and Susceptibilities to Intracranial Aneurysm in Chinese Population
Source: Biomed Res Int. 2021 Jan 16;2021:8865601. doi: 10.1155/2021/8865601 (PMC7826207; doi:10.1155/2021/8865601)
Supplement: Supplementary Materials — Table S1: PCR primers designed for SNPs. Table S2: univariate logistic regression analysis of associations between inflammatory cytokine gene polymorphisms and risk of IA in Chinese population. Table S3: univariate logistic regression analysis of associations between inflammatory cytokine gene polymorphisms and risk of single IA in Chinese population. Table S4: univariate logistic regression analysis of associations between inflammatory cytokine gene polymorphisms and risk of multiple IAs in Chinese population. [file 8865601.f1.zip › Table S3 (1).docx]

| **Table S3.** Univariate logistic regression analysis of associations between inflammatory cytokine gene polymorphisms and risk of single intracranial aneurysm in Chinese population | | | | | | | | | | |
| --- | --- | --- | --- | --- | --- | --- | --- | --- | --- | --- |
| Gene | SNPs | Genotype* | | Dominant model | | Recessive model | | Additive model | | *P*_HWE_^†^ |
|  |  | Case (n) | Control (n) | OR (95% CI) | *P* | OR (95% CI) | *P* | OR (95% CI) | *P* |  |
| *IL-1A* | rs17561 | 224/23/1 | 318/65/1 | 0.52(0.31-0.85) | 0.009 | 1.55(0.10-24.91) | 0.757 | 0.55(0.34-0.88) | 0.014 | 0.218 |
| *IL-1B* | rs1143627 | 48/126/74 | 93/185/106 | 1.33(0.90-1.97) | 0.152 | 1.12(0.78-1.59) | 0.543 | 1.15(0.92-1.44) | 0.220 | 0.489 |
|  | rs16944 | 44/128/76 | 88/187/109 | 1.38(0.92-2.06) | 0.119 | 1.12(0.79-1.58) | 0.542 | 1.16(0.93-1.46) | 0.195 | 0.651 |
|  | rs1143623 | 91/115/42 | 144/179/61 | 1.04(0.74-1.44) | 0.838 | 1.08(0.70-1.66) | 0.727 | 1.04(0.83-1.30) | 0.745 | 0.666 |
|  | rs1143630 | 8/68/172 | 13/102/269 | 1.05(0.43-2.57) | 0.913 | 0.97(0.68-1.37) | 0.852 | 0.98(0.73-1.32) | 0.902 | 0.391 |
|  | rs2853550 | 0/35/213 | 5/75/304 | - | - | 1.60(1.04-2.47) | 0.033 | 1.65(1.09-2.50) | 0.018 | 0.878 |
|  | rs3136558 | 99/115/34 | 171/172/41 | 1.21(0.87-1.67) | 0.253 | 1.33(0.82-2.16) | 0.251 | 1.18(0.93-1.50) | 0.163 | 0.817 |
| *IL6* | rs1800795 | 0/0/248 | 0/0/384 | - | - | - | - | - | - | - |
|  | rs1800796 | 10/84/154 | 18/141/225 | 1.17(0.53-2.58) | 0.696 | 1.16(0.84-1.61) | 0.380 | 1.13(0.86-1.50) | 0.378 | 0.491 |
| *IL12B* | rs3181216 | 136/97/15 | 191/155/38 | 0.82(0.59-1.12) | 0.211 | 0.59(0.32-1.09) | 0.091 | 0.80(0.62-1.03) | 0.088 | 0.429 |
|  | rs3212227 | 77/116/55 | 107/195/82 | 0.86(0.61-1.22) | 0.390 | 1.05(0.71-1.55) | 0.806 | 0.95(0.76-1.20) | 0.683 | 0.696 |
|  | rs1003199 | 84/130/34 | 148/190/46 | 1.10(0.79-1.53) | 0.568 | 0.94(0.57-1.54) | 0.793 | 1.04(0.81-1.32) | 0.769 | 0.205 |
|  | rs2195940 | 226/20/2 | 341/41/2 | 0.77(0.45-1.33) | 0.348 | 1.55(0.22-11.10) | 0.661 | 0.83(0.50-1.36) | 0.451 | 0.528 |
| *TNF-α* | rs1800629 | 218/29/1 | 342/41/1 | 1.12(0.68-1.84) | 0.654 | 1.55(0.10-24.91) | 0.757 | 1.13(0.70-1.81) | 0.630 | 0.844 |
|  | rs1799724 | 187/60/1 | 298/77/9 | 1.13(0.78-1.65) | 0.523 | 0.17(0.02-1.34) | 0.092 | 1.01(0.72-1.42) | 0.945 | 0.141 |
|  | rs1799964 | 168/75/5 | 252/112/20 | 0.91(0.65-1.28) | 0.582 | 0.37(0.14-1.01) | 0.053 | 0.84(0.63-1.13) | 0.245 | 0.111 |
| SNPs, single nucleotide polymorphisms; OR, odds ratio; CI, confidence interval; HWE, Hardy-Weinberg equilibrium. | | | | | | | | | | |
| *Genotype presented as wild type/heterozygous/homozygous, † HWE *P* value for the control group. | | | | | | | | | | |
