# Supplementary material for: Associations between Inflammatory Cytokine Gene Polymorphisms and Susceptibilities to Intracranial Aneurysm in Chinese Population
Source: Biomed Res Int. 2021 Jan 16;2021:8865601. doi: 10.1155/2021/8865601 (PMC7826207; doi:10.1155/2021/8865601)
Supplement: Supplementary Materials — Table S1: PCR primers designed for SNPs. Table S2: univariate logistic regression analysis of associations between inflammatory cytokine gene polymorphisms and risk of IA in Chinese population. Table S3: univariate logistic regression analysis of associations between inflammatory cytokine gene polymorphisms and risk of single IA in Chinese population. Table S4: univariate logistic regression analysis of associations between inflammatory cytokine gene polymorphisms and risk of multiple IAs in Chinese population. [file 8865601.f1.zip › Table S4 (1).docx]

| **Table S4.** Univariate logistic regression analysis of associations between inflammatory cytokine gene polymorphisms and risk of multiple intracranial aneurysm in Chinese population | | | | | | | | | | |
| --- | --- | --- | --- | --- | --- | --- | --- | --- | --- | --- |
| Gene | SNPs | Genotype* | | Dominant model | | Recessive model | | Additive model | | *P*_HWE_^†^ |
|  |  | Case (n) | Control (n) | OR (95% CI) | *P* | OR (95% CI) | *P* | OR (95% CI) | *P* |  |
| *IL-1A* | rs17561 | 117/18/1 | 318/65/1 | 0.78(0.45-1.36) | 0.384 | 2.84(0.18-45.67) | 0.462 | 0.82(0.49-1.40) | 0.474 | 0.218 |
| *IL-1B* | rs1143627 | 29/73/34 | 93/185/106 | 1.18(0.74-1.89) | 0.494 | 0.87(0.56-1.37) | 0.556 | 1.01(0.76-1.33) | 0.967 | 0.489 |
|  | rs16944 | 27/68/41 | 88/187/109 | 1.20(0.74-1.95) | 0.460 | 1.09(0.71-1.67) | 0.697 | 1.10(0.84-1.45) | 0.496 | 0.651 |
|  | rs1143623 | 43/70/23 | 144/179/61 | 1.30(0.96-1.97) | 0.220 | 1.08(0.64-1.82) | 0.780 | 1.15(0.87-1.53) | 0.319 | 0.666 |
|  | rs1143630 | 4/40/92 | 13/102/269 | 1.16(0.37-3.61) | 0.802 | 0.89(0.59-1.36) | 0.601 | 0.94(0.65-1.34) | 0.715 | 0.391 |
|  | rs2853550 | 0/23/113 | 5/75/304 | - | - | 1.29(0.78-2.16) | 0.325 | 1.35(0.83-2.20) | 0.224 | 0.878 |
|  | rs3136558 | 61/61/14 | 171/172/41 | 0.99(0.67-1.46) | 0.948 | 0.96(0.51-1.82) | 0.901 | 0.98(0.73-1.32) | 0.915 | 0.817 |
| *IL6* | rs1800795 | 0/0/136 | 0/0/384 | - | - | - | - | - | - | - |
|  | rs1800796 | 7/53/76 | 18/141/225 | 0.91(0.37-2.22) | 0.830 | 0.90(0.60-1.33) | 0.582 | 0.91(0.66-1.27) | 0.589 | 0.491 |
| *IL12B* | rs3181216 | 71/55/10 | 191/155/38 | 0.91(0.61-1.34) | 0.621 | 0.72(0.35-1.49) | 0.380 | 0.89(0.66-1.20) | 0.443 | 0.429 |
|  | rs3212227 | 28/75/33 | 107/195/82 | 1.49(0.93-2.39) | 0.098 | 1.18(0.74-1.87) | 0.482 | 1.24(0.93-1.64) | 0.141 | 0.696 |
|  | rs1003199 | 47/78/11 | 148/190/46 | 1.45(0.96-2.21) | 0.081 | 1.05(0.58-1.90) | 0.873 | 1.23(0.91-1.66) | 0.172 | 0.205 |
|  | rs2195940 | 128/8/0 | 341/41/2 | 0.50(0.23-1.08) | 0.078 | - | - | 0.49(0.23-1.06) | 0.069 | 0.528 |
| *TNF-α* | rs1800629 | 119/17/0 | 342/41/1 | 1.16(0.64-2.12) | 0.622 | - | - | 1.13(0.63-2.03) | 0.689 | 0.844 |
|  | rs1799724 | 106/26/4 | 298/77/9 | 0.98(0.61-1.57) | 0.935 | 1.26(0.38-4.17) | 0.702 | 1.01(0.68-1.51) | 0.957 | 0.141 |
|  | rs1799964 | 104/27/5 | 252/112/20 | 0.59(0.38-0.92) | 0.020 | 0.70(0.26-1.89) | 0.475 | 0.66(0.46-0.96) | 0.032 | 0.111 |
| SNPs, single nucleotide polymorphisms; OR, odds ratio; CI, confidence interval; HWE, Hardy-Weinberg equilibrium. | | | | | | | | | | |
| *Genotype presented as wild type/heterozygous/homozygous, † HWE *P* value for the control group. | | | | | | | | | | |
